# New Ecological Role of Seaweed Secondary Metabolites as Autotoxic and Allelopathic

**DOI:** 10.3389/fpls.2020.00347

**Published:** 2020-05-25

**Authors:** Daniela Bueno Sudatti, Heitor Monteiro Duarte, Angélica Ribeiro Soares, Leonardo Tavares Salgado, Renato Crespo Pereira

**Affiliations:** ^1^Departamento de Biologia Marinha, Instituto de Biologia, Universidade Federal Fluminense, Niterói, Brazil; ^2^Grupo de Produtos Naturais de Organismos Aquáticos (GPNOA), Núcleo de Estudos em Ecologia e Desenvolvimento Sócio-ambiental de Macaé, Universidade Federal do Rio de Janeiro, Macaé, Brazil; ^3^Instituto de Pesquisas Jardim Botânico do Rio de Janeiro, Rio de Janeiro, Brazil

**Keywords:** chemical defense, photosynthesis inhibition, crossed experiments, elatol, obtusol

## Abstract

Allelopathy and autotoxicity are well-known biological processes in angiosperms but are very little explored or even unknown in seaweeds. In this study, extract and major pure compounds from two distinct populations of the red seaweed *Laurencia dendroidea* were investigated to evaluate the effect of autotoxicity through auto- and crossed experiments under laboratory conditions, using chlorophyll fluorescence imaging to measure inhibition of photosynthesis (Φ_PSII_) as a variable response. Individuals of *L. dendroidea* from Azeda beach were inhibited by their own extract (IC_50_ = 219 μg/ml) and the major compound elatol (IC_50_ = 87 μg/ml); both chemicals also inhibited this seaweed species from Forno beach (IC_50_ = 194 μg/ml for the extract and IC_50_ = 277 μg/ml for elatol). By contrast, the extract of *L. dendroidea* from Forno and its major compound obtusol showed no inhibitory effect in individuals of both populations; but obtusol was insoluble to be tested at higher concentrations, which could be active as observed for elatol. The Azeda population displayed higher susceptibility to the Azeda extract and to elatol, manifested on the first day, unlike Forno individuals, in which the effect was only detected on the second day; and inhibition of Φ_PSII_ was more pronounced at apical than basal portions of the thalli of *L. dendroidea.* This first finding of seaweed autotoxicity and allelopathic effects revealed the potential of the chemistry of secondary metabolites for intra- and inter-populational interactions, and for structuring seaweed populations.

## Introduction

Allelopathy is an ecological strategy employed by plants that release chemicals to increase interspecific and intraspecific competitive ability. Allelopathy can also determine the patterns of spatial distribution of organisms and the structuring of a community (Kato-Noguchi et al., 2018). Secondary metabolites produced by marine organisms can act as defense against competitors, one of the processes within the concept of allelopathy (Paul et al., 2011). These chemicals from seaweeds have been shown to inhibit growth of other seaweed species (Kim et al., 2004; Kumler, 2017), as well as seagrass (Raniello et al., 2007), bacteria (Hellio et al., 2001; Lam and Harder, 2007), and diatom (Lu et al., 2011); to cause bleaching in corals (Rasher et al., 2011); and to affect the survivorship and settlement of coral larvae (Fong et al., 2019).

Allelopathy among terrestrial plants includes intraspecific interactions that are important in controlling population density and are hypothesized to minimize resource competition, enhancing population viability (Gomes et al., 2017). The same compounds can act in both allelopathy interactions, interspecific and intraspecific (Perry et al., 2005). Several interspecific (Gomes et al., 2017; Silva et al., 2017) and intraspecific – also designed as autotoxicity and/or autoinhibition (Singh et al., 1999; Kato-Noguchi et al., 2018) – allelopathic interactions have been described in terrestrial plants, but these interactions are less studied among seaweed species (but see Kumler, 2017).

The red seaweeds belonging to the *Laurencia* complex are prolific producers of secondary metabolites: among the 430 species of the genus, more than 1,000 compounds, mainly halogenated terpenes, were described as having both pharmacological and ecological activities (Harizani et al., 2016). Secondary metabolites from *Laurencia* species exhibit deterrence against consumption by sea urchins (Pereira et al., 2003), reef fishes (Hay et al., 1987), and snails (Granado and Caballero, 1995) and also inhibit the settlement of fouling organisms (Da Gama et al., 2002) and marine bacteria (Vairappan et al., 2009). Within the *Laurencia* complex, the species *Laurencia dendroidea* was described as a producer of powerful halogenated sesquiterpenes that actively play ecological roles such as anti-herbivory (Pereira et al., 2003) and anti-fouling (Da Gama et al., 2002; Paradas et al., 2010). In *L. dendroidea*, a distinct cell death event was morphologically marked by a sudden rupture of the membrane of the main organelle storing the halogenated compounds, which subsequently led to vacuole membrane retraction and chloroplast degradation (Salgado et al., 2008), thus raising the possibility of autotoxicity. Furthermore, populations of *L. dendroidea* display different chemical profiles (Machado et al., 2016), suggesting selective pressure for a differential defense strategy that can affect density-dependent mortality process among the population. However, to our knowledge, intraspecific allelopathic (autotoxicity) interactions among seaweeds have not been documented. Thus, here, we addressed the autotoxicity in seaweed according to the following questions: (a) Do *L. dendroidea* secondary metabolites promote autotoxicity? (b) Is there any specificity inhibitory effect according to secondary metabolites in each population (auto- and cross-effect)?

## Materials and Methods

### Sample Collection

Two different populations of *L. dendroidea* from Rio de Janeiro state, found at Forno Beach, Arraial do Cabo (22°58′003.3″S, 42°00′56.2″W), and Azeda Beach, Armação dos Búzios (22°44′33.6″S, 41°52′055.6″W), were used in this study. Specimens of *L. dendroidea* were collected at depths of 1–2 m. Collected organisms were used for secondary metabolite purification or for carrying out the autotoxicity bioassays. Prior to bioassays, specimens were acclimated to laboratory conditions, that is, incubated in seawater at 22 ± 2°C, with salinity of 32 ± 1% and irradiance of 80 μmol photons m^–2^ s^–1^ (provided by cool-white fluorescent lamps with a 12:12-h light:dark cycle), with aeration for 2 days. Voucher specimens were deposited at the Herbarium of the Rio de Janeiro Federal University, Brazil (Forno beach: RFA 36141, Azeda beach: RFA 38846).

### Chemical Extraction and Secondary Metabolite Purification

**FIGURE 1:**
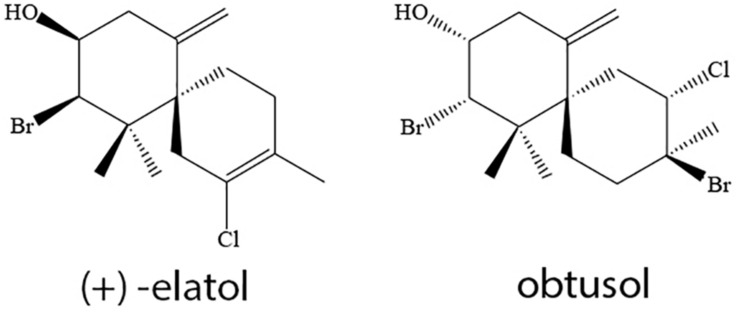
Major compounds produced by specimens of *Laurencia dendroidea* from Azeda (elatol) and Forno (obtusol) populations.

To obtain the extracts of both populations of *L. dendroidea* (from Azeda, AE, and Forno, FE), collected specimens were initially washed with seawater, dried at room temperature, and further extracted three times in dichloromethane (Tedia) during a 72-h period. Extracts were filtered and dried by rotatory evaporation. These populations were selected because they seem to be chemotypes of *L. dendroidea*, according to their secondary metabolite profiles previously observed by our research group (Machado et al., 2016). The halogenated sesquiterpenes (+)-elatol (mentioned simply as elatol) and obtusol (Figure 1) were identified as major compounds in Azeda and Forno populations, respectively. Furthermore, GC–MS profiles in algae from Forno and Azeda populations indicate that elatol and obtusol are unique in each of these selected populations (Machado et al., 2016).

The major compounds of both extract were obtained and identified as described previously (Machado et al., 2016). Extracts were first separated by silica gel column chromatography eluted in a step gradient of organic solvents (Hexane, CH_2_Cl_2_, AcOECt, and MeOH), resulting in several fractions. Fraction purification was guided by TLC (Merck Al TLC 20 × 20-cm silica gel 60F254) and submitted to spectroscopic analyses of ^1^H and ^1^C NMR, nuclear magnetic resonance. Determination of halogenated sesquiterpenes (+)-elatol (Sims et al., 1974; Martin et al., 1989; König and Wright, 1997) and obtusol (González et al., 1979; Wessels et al., 2000) was done by comparing the spectroscopic data with those reported in literature (see Supplementary Material S1). The obtained extracts and major compounds were used in allelopathic bioassays and related analysis.

The autotoxicity of *L. dendroidea* extracts and their major metabolites was accessed by measuring their effects on the photosystem II (PSII) yield of specimens from Azeda and Forno. The inhibitory effect of the extracts and major compounds on the effective quantum yield of PSII (Φ_PSII_) was determined in both populations in auto- and cross-assays; thus, interpopulational and intrapopulational effects were evaluated. When inhibition was observed, IC_50_ was determined. IC_50_ values were obtained by regression analysis using a sigmoidal logistic equation with three parameters: *y* = *a*/[1 + (*x*/IC_50_)*^*b*^*], where *a* is the highest effect in ΦPSII value, *b* is the exponential decay constant, and IC_50_ is the concentration at which the effect of ΦPSII reaches 50% from its maximal value.

The *L*. *dendroidea* extracts and isolated compounds were dissolved in dimethyl sulfoxide (DMSO) and diluted into sterilized seawater to yield a final concentration of 2% of DMSO. The nontoxic DMSO concentration was determined before the experiments. For this, algal thalli were incubated in serial DMSO concentrations, and the effective quantum yield of PSII (see below) was measured in the same way as in allelopathic assays (results are present as Supplementary Material S2). Aiming to compare the efficacy of extracts and major compounds, we used the natural concentrations of elatol (∼50% of extract) and obtusol (∼18% of extract) according to a previous study (Machado et al., 2016). To obtain the dose–response curve for extracts and compounds, a range of serially diluted concentrations was obtained, beginning from each initial concentration (FE: 25–125 μg/ml; AE: 10–275 μg/ml; elatol: 50–250 μg/ml; obtusol: 50–150 μg/ml). The solutions were then poured into a 24-well flat-bottom plate where each well (3.5-ml total well capacity) received one fragment (1.5-cm length) of *L. dendroidea*. Fragments were removed from fronds 2 days before to minimize stress (e.g., chemical defense induction due to damage; Sudatti et al., 2008) and to allow acclimation. Four replicates of *L. dendroidea* fragments were used for each concentration tested. Lastly, the plates were placed under the chlorophyll fluorescence image system (see below) to measure two successive light/dark cycles (12 h/12 h).

### Chlorophyll Fluorescence

Inhibition of photosynthesis is a frequent mode of action in allelopathic interactions of primary producers (Gross, 2003). Thus, to verify the inhibitory effect of extracts and major compounds of *L. dendroidea*, our approach was to use chlorophyll fluorescence imaging to produce time series of the effective quantum yield of PSII (Φ_PSII_). Determination of the quantum yield of PSII (Φ_PSII_) was undertaken using an imaging system developed at NUPEM/UFRJ, previously used to measure this activity in *L. dendroidea* (Sudatti et al., 2016) and described previously (Gross, 2003). The photosynthetic and excitation lights were provided by four arrays of 36 blue light-emitting diodes (μ = 470 nm, maximum power 432 W), the intensity of which was micro-controlled by pulse-width modulation at a frequency of 1,200 Hz. During the experiments, the light intensity was kept constant at 200 μmol photons m^–2^ s^–1^. Chlorophyll fluorescence was selectively detected by a Peltier-cooled digital camera Alta U6 (Apogee Inc., United States), equipped with a CCD sensor of 1,024 × 1,024 pixels and 16-bit digitalization. A μ < 665 filter RG-655 Schott (Mainz, Germany) was attached to the camera objective (60-mm macro lens, Nikkor, Nikon, United States). Images of chlorophyll fluorescence were recorded and processed on a PC by customized software written in Visual C++. The Φ_PSII_ was recorded according to the saturating light pulse method (Genty et al., 1989; Schreiber et al., 1994). First, an image of the sample steady-state fluorescence (*iF*) under constant light intensity was recorded. After that, the algal samples were exposed to a saturating light pulse (intensity: ∼2,500 μmol photons m^–2^ s^–1^, duration: 800 ms). The last 200 ms of this pulse was used to integrate the maximal fluorescence signal of the second image (*iFm*). Both *iF* and *iFm* were corrected by dividing them by the pixel mean of an internal fluorescence standard (Walz, Germany) placed close to the sample. Images of *i*Φ_PSII_ were recorded every 10 min and calculated as *i*Φ_PSII_ = (*iFm* – *iF*)/(*iFm* ⋅ *i*Φ_PSII_). Posterior image processing to calculate the pixel average of each individual sample was conducted with the software ImageJ (Abramoff et al., 2004).

## Results

**FIGURE 2:**
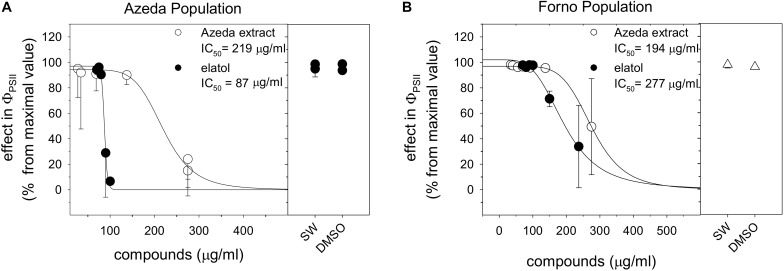
Toxicity of *Laurencia dendroidea* extract and the major compound on individuals of this seaweed from Azeda and Forno populations. Toxicity expressed as inhibition of the effective quantum yield of photosystem II (Φ_PSII_) in auto- **(A)** and cross-assays **(B)**. Seawater (SW) and DMSO in SW were used as null control and control, respectively. The graph shows Φ_PSII_ means and confidence intervals (Student’s *t* distribution, *p* < 0.05). *N* = 4 per tested concentration.

Azeda extract and elatol inhibited the effective quantum yield of PSII (Φ_PSII_) in auto- and cross-assays. Individuals of *L. dendroidea* from the Azeda population were inhibited by their own extract, AE (IC_50_ = 219 μg/ml, Figure 2A) and major compound elatol (IC_50_ = 87 μg/ml, Figure 2A). Individuals of *L. dendroidea* from Forno were also inhibited by AE (IC_50_ = 194 μg/ml, Figure 2B) and elatol (IC_50_ = 277 μg/ml, Figure 2B). The negative signal effects of AE started at similar concentrations on both populations of *L. dendroidea* (around 125 μg/ml). But the negative effect of elatol was stronger in the Azeda than Forno population.

Extract of *L. dendroidea* from Forno (FE) and obtusol showed no inhibitory effect on Φ_PSII_ in individuals of this seaweed from both populations at tested concentrations (Supplementary Material S3). FE and the major compound of the Forno population (obtusol) were insoluble above 100 and 125 μg/ml, respectively. Concentrations of DMSO higher than 2%, which may have allowed increased solubility, were toxic to individuals of both populations (Supplementary Material S2).

**FIGURE 3:**
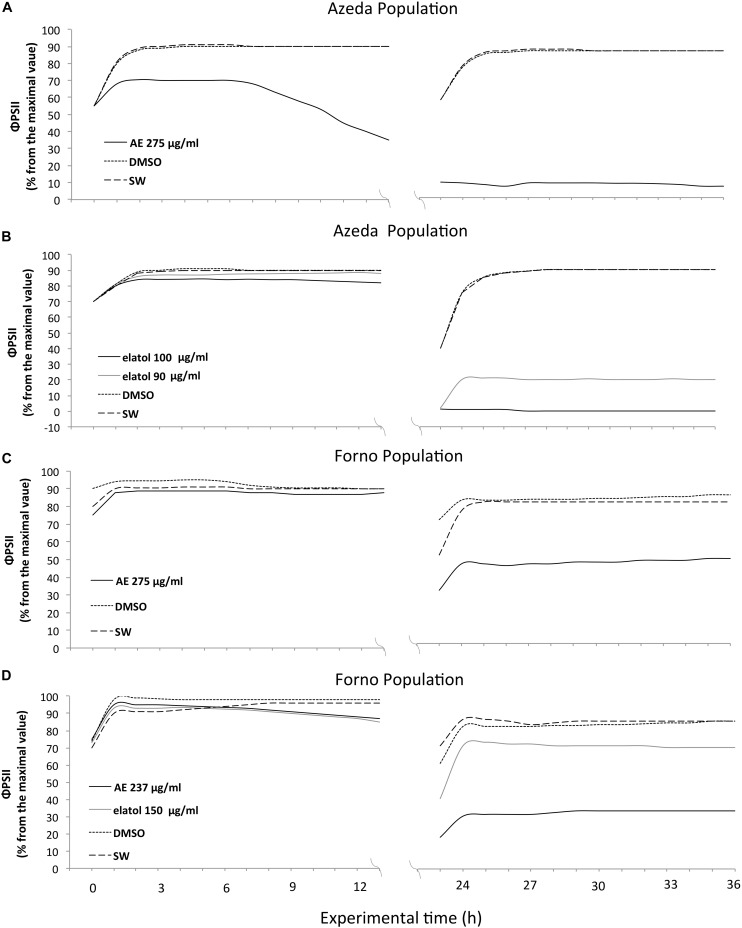
Higher susceptibility of individuals from the Azeda population to the Azeda extract and elatol than of individuals from the Forno population. Azeda Φ_PSII_ inhibition to the Azeda extract occurred on the first day **(A)**, while for Forno, it occurred on the second day **(C)**. Also, lower elatol concentrations were necessary to inhibit Φ_PSII_ in Azeda **(B)** individuals of *Laurencia dendroidea* than those from the Forno population **(D)**.

The temporal course of Φ_PSII_ was analyzed on the assays where inhibitory effect was observed. The Azeda population of *L. dendroidea* displayed higher susceptibility to both AE and elatol than the Forno population, albeit in distinct ways for each treatment. The AE caused faster inhibition in Azeda *L. dendroidea* (*i*Φ_PSII_ = 60% since the first hours of the experiment at 275 μg/ml; Figure 3A) than in Forno (*i*Φ_PSII_ = 45%, only after 24 h at 275 μg/ml; Figure 3C) individuals of this seaweed, whereas elatol had lower inhibitory concentrations for the Azeda (*i*Φ_PSII_ = 15%, after 24 h at 90 μg/ml and *i*Φ_PSII_ = 0%, after 24 h at 100 μg/ml; Figure 3B) than for the Forno population (*i*Φ_PSII_ = 65%, after 24 h at 150 μg/ml; Figure 3D). Thus, the effect of the AE on the Azeda population of *L. dendroidea* was manifested in the first day of treatment (Figure 3A), whereas the effect of elatol was only detected on the second day (Figure 3B), as well as on the Forno population (Figures 3C, D).

**FIGURE 4:**
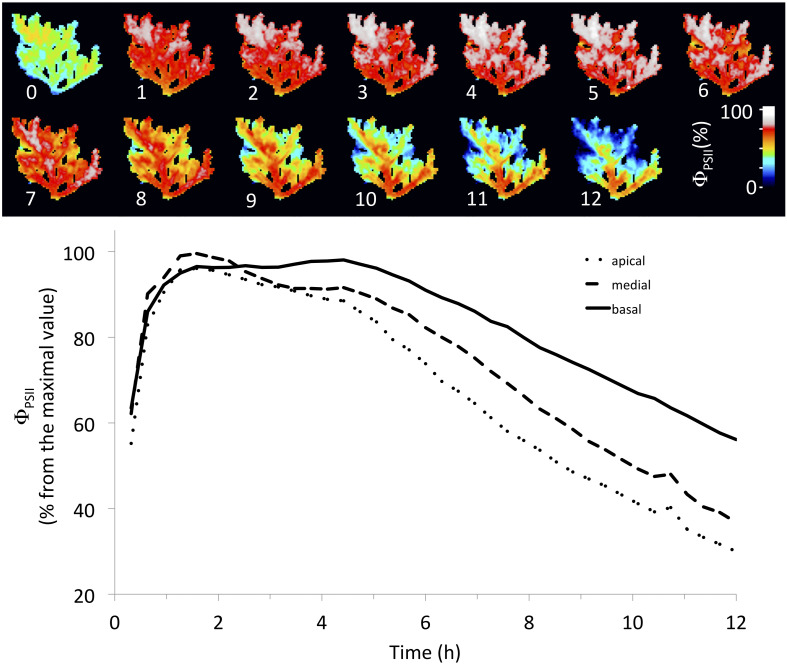
Stronger inhibitory effect of extract and metabolite on apical than basal portions of the algal thalli. The upper figure shows the spatial–temporal effect of AE in individuals of the Azeda population. The lower figure shows the decrease of effective quantum yield of photosystem II (Φ_PSII_) during the time in apical, medial, and basal portions of the algal thalli.

There was a spatial heterogeneity on the inhibition of Φ_PSII_, which was more pronounced at apical than basal parts of the thalli (Table 1). After 24 h of experiment, apical parts had a Φ_PSII_ around 30% from the maximal value, while basal parts had Φ_PSII_ at 60% (Figure 4), mainly when toxicity was observed during the light period of the first day (Figure 3A). However, this pattern was less evident when the toxicity was observed at the second day, probably because its effect started during the dark period.

**TABLE 1:** Repeated-measures ANOVA evaluating the spatial (portion) and temporal inhibitory effects (Φ_PSII_) of AE on apical, medial, and basal portions of the algal thalli.

Factor	Variation source	df	Sum square	Mean square	*F*	*p*
Φ_PSII_	Time	37	0.195	0.005	5.603	<0.001
	Portion	2	0.209	0.104	111.329	<0.001
	Time vs. portion	2	0.088	0.001	1.266	<0.104

## Discussion

In this work, we investigated whether intraspecific allelopathy (autotoxicity), already described in terrestrial plants, also occurs in seaweeds. By crossed laboratory experiments, we demonstrated that the extract and pure compound of one of the seaweed chemotypes were indeed toxic for both chemotypes of the red seaweed *L. dendroidea*. However, susceptibility varied between the chemotypes, in terms of both inhibitory concentration of photosynthesis and time of onset of the effect. This was true both for the extract of *L. dendroidea* from the Azeda population (AE) and for its major compound secondary metabolite, elatol. There was also a difference in susceptibility between the apical and basal portions of the algal thalli.

The autotoxicity of the AE and elatol can provide an explanation for the energetic investment in storage structures (*corps in cerise*) and in transport mechanisms to the apoplastic space. These results are consistent with a hypothesis proposed previously (Salgado et al., 2008; Sudatti et al., 2016), according to which the traffic of secondary metabolites inside vesicles (*corps in cerise*) could allow increased concentration of the metabolite on the surface without promoting toxicity for *L. dendroidea* cells and also permitting the regulation of metabolite exudation. In fact, transport mechanisms can reduce the cytosolic concentration of active compounds, resulting in a decrease in cytotoxicity (Shitan, 2016). Likewise, compartmentalization of other secondary metabolites, such as phlorotannins in physodes of brown seaweeds (Ank et al., 2014), furanones in gland cells in red seaweed *Delisea pulchra* (Maximilien et al., 1998; Steinberg and de Nys, 2002), and crambescins/crambescidins in spherulous in *Crambe crambe* sponge cells (Ternon et al., 2016), could also contribute to autotoxicity avoidance.

Differential susceptibility between populations indicated a more pronounced intrapopulational than interpopulational inhibition: AE was effective from the first day in the Azeda samples, but only on the second day for Forno samples. Moreover, the levels of elatol that promoted Φ_PSII_ decrease were lower for the Azeda than for the Forno population of *L. dendroidea*. In terrestrial plants, autotoxicity is a strategy to control population density, constrain inbreeding, and promote genetic variability, favoring the introduction of foreign genotypes (Singh et al., 1999). Although we do not have information about *L. dendroidea* population density and spatial distribution, we can also presuppose this similar effect for this red seaweed. Higher population density in seaweeds mainly affects light availability, as a result of shading (for a review, see Edwards and Connell, 2012). Thus, higher density can decrease growth of seaweeds, and autotoxicity may play an important role in structuring population by density control.

Moreover, although information on the genetic structure of Brazilian *L. dendroidea* populations is lacking, it is implied by the qualitative and quantitative variations in secondary metabolites (Machado et al., 2016), which are the basis of the chemotypes, such as the two populations studied. A similar variation was also reported for other *Laurencia* species (Howard et al., 1980; Masuda et al., 1997; Abe et al., 1999). Thus, allelopathy through chemotypes can mediate both intrapopulation competition (population density control) and genetic flux. Indeed, the existence of populations that present either elatol or obtusol as well as populations with intermediate chemical profiles (see Machado et al., 2016) is consistent with the hypothesis of existence of genetic flux.

Several physiological and biochemical parameters might be considered as effective tools to quantify susceptibility to allelochemical inhibitors (Bouhaouel et al., 2018). Here, we imaged the quantum yield of PSII (Φ_PSII_), which made it possible to take both temporal and spatial measures of photosynthetic performance for estimating allelopathic effect. The latter allowed us to observe that both extract of individuals of *L. dendroidea* from the Azeda population and its major compound elatol inhibited initially the apical portions and posteriorly the basal portions of the algal thalli. Cells in the apical portions are younger (Honkanen and Jormalainen, 2002) than those in the basal (main axis) portions and thus could be more susceptible to autotoxic secondary metabolites, perhaps due to their having thinner cell walls and higher photosynthetic activity, indicating a trade-off between susceptibility to chemical defense and growth. Similarly, the apical portions of seaweeds are more vulnerable to herbivory due to lower levels of chemical defense (Hay et al., 1988).

Elatol was found only in the Azeda population, while obtusol was present only in the Forno population (see section “Materials and Methods”), and thus, our experiments were designed to compare auto- and cross-population allelopathic effects. The Forno extract and obtusol displayed no autotoxicity for either population at the tested concentrations. As above those concentrations they tended to precipitate, solubility may have limited their ability to act as toxic compounds. This result might differentiate the autotoxicity effects within populations, like Azeda, that produce elatol from those that produce obtusol. Consistent with our results, higher levels of obtusol were necessary to affect herbivory, fish larval toxicity (Granado and Caballero, 1995), and antibacterial activity (Vairappan et al., 2009). In those assays, obtusol was incorporated in a matrix (e.g., palatable seaweed and paper disc), and thus, solubility was not an issue. Conversely, elatol has potent inhibitory effects in several ecological interactions (Granado and Caballero, 1995; Da Gama et al., 2002), but concentration and target organisms determine the efficacy of this natural product, for example, as antifouling (De Nys et al., 1996) and antiherbivory (Sudatti et al., 2018). On the other hand, the defensive role of extracts may not depend exclusively on a major compound, because a mixture of allelochemicals may have a positive effect, even while pure compounds do not (Blum et al., 1999). Thus, mixtures of some allelochemicals (e.g., phenolic acids) can possess allelopathic activity even though concentrations of individual compounds are significantly below their inhibitory levels (Blum, 1996). This effect could underlie the fact that the extract of *L. dendroidea* from Azeda acted earlier (on the first day) than did pure elatol (which had an effect only on the second day). This highlights the importance of working with conditions that mimic, as closely as possible, the ecological reality.

The inhibitory effect of AE and elatol on Φ_PSII_ of *L. dendroidea* in auto- and cross-assays indicates, for the first time, autotoxicity in seaweeds. These findings widen the possible adaptive value of seaweed secondary metabolites. Intraspecific allelopathy or autotoxicity is well described in terrestrial plants, in which it has both ecological (Blum, 1996) and crop implications (Aslam et al., 2017). Similarly, insights about autotoxicity contribute to understanding natural seaweed population density control, which may help to define adequate seaweed culture conditions. Further studies should investigate the autotoxic effects on other physiologic parameters of seaweed population health, such as spore germination or growth. Moreover, the effect of solubility on secondary metabolite availability suggests that contact experiments might be performed to better elucidate the autotoxic and allelopathic action mechanisms in *L. dendroidea*. Indeed, interspecific allelopathy involving seaweed mediated by contact has already been described (Raniello et al., 2007; Lu et al., 2011; Kumler, 2017).

Furthermore, the use of PSII imaging allowed us to observe the photosynthetic spatial–temporal dynamics of several algal thalli incubated with secondary metabolites and thus to evaluate the differential susceptibility of portions of the seaweed thallus. The technique was seen as a sensitive and robust method that allows fast acquisition of data from a large sample, while providing both spatial and temporal information. This methodology might be a powerful tool to study autotoxicity and allelopathy in a variety of seaweeds and other marine photosynthetic organisms.

The molecular mechanisms that lead to elatol autotoxicity are not yet understood, especially as concerns the higher intrapopulational toxicity. Since the main parameter used to demonstrate elatol autotoxicity is the photosynthetic quantum yield, looking into the chloroplast metabolism is necessary to understand this allelopathic effect on *L. dendroidea* populations. A correlation between the elatol metabolism and chloroplast activity was first demonstrated (Sudatti et al., 2016), which revealed that tissue elatol concentration is higher when algae experiences low Φ_PSII_ caused by dark conditions. In the present work, direct evidence demonstrated that higher intracellular elatol concentrations could decrease the Φ_PSII_.

Concerning the metabolic regulation, it was revealed that the secondary metabolism pathways in plastids are feedback regulated. For example, the downregulation of the plastidial DXP pathway in *Populus trichocarpa*, in which the enzyme deoxyxylulose-5-phosphate synthase (DXS) was inhibited by two isoprenoid precursors synthetized by the same pathway, the isopentenyl diphosphate and the dimethylallyl diphosphate (Banerjee et al., 2013). Concerning *L. dendroidea*, it is important to mention that the DXP/MEP pathways are involved in their secondary metabolism (Oliveira et al., 2015), which has been shown to be upregulated and downregulated at both cellular and molecular levels (Paradas et al., 2010; Oliveira et al., 2012).

A direct connection between photosynthetic activity and secondary metabolism was first described in cyanobacterial (Okada and Hase, 2005) and thylakoid ferredoxin I (PetF) transfers electrons from the electron transport chain to 4-hydr oxy-3-methylbut-2-enyl diphosphate synthase (HDS), an enzyme from the DXP pathway activated by light condition (Bouvier et al., 2005). Interestingly, it was already reported that elatol interferes in mitochondrial activity, once it specifically increases ROS through the electron transport chain at mitochondria in *Trypanosoma cruzi* protozoan (Desoti et al., 2012).

Thus, following this concept, it is feasible to hypothesize that algae exposition to the extract and elatol may interfere with their intracellular levels, causing downregulation of the DXP pathway, reduction of HDS expression, and lower electron output rates from PetF, which finally would interfere with photosynthetic activity. Once *L. dendroidea* from the Forno population synthesized obtusol, an elatol-similar structure, perhaps the downregulation process would be less intense in this population, resulting in a higher tolerance (IC_50_ = 194 μg/ml). However, the intricate chemoactivity of these algal metabolites should be better investigated in order to understand their mechanisms of autotoxicity.

## Data Availability Statement

The datasets generated for this study are available on request to the corresponding author.

## Author Contributions

DS, HD, and AS conceived the ideas, designed the methodology, collected, and analyzed the data. DS, HD, AS, LS, and RP led the writing of the manuscript. RP and LS acquired the funding.

## Conflict of Interest

The authors declare that the research was conducted in the absence of any commercial or financial relationships that could be construed as a potential conflict of interest.
