# Supplementary figures and images for: New Ecological Role of Seaweed Secondary Metabolites as Autotoxic and Allelopathic
Source: Front Plant Sci. 2020 May 25;11:347. doi: 10.3389/fpls.2020.00347 (PMC7261924; doi:10.3389/fpls.2020.00347)

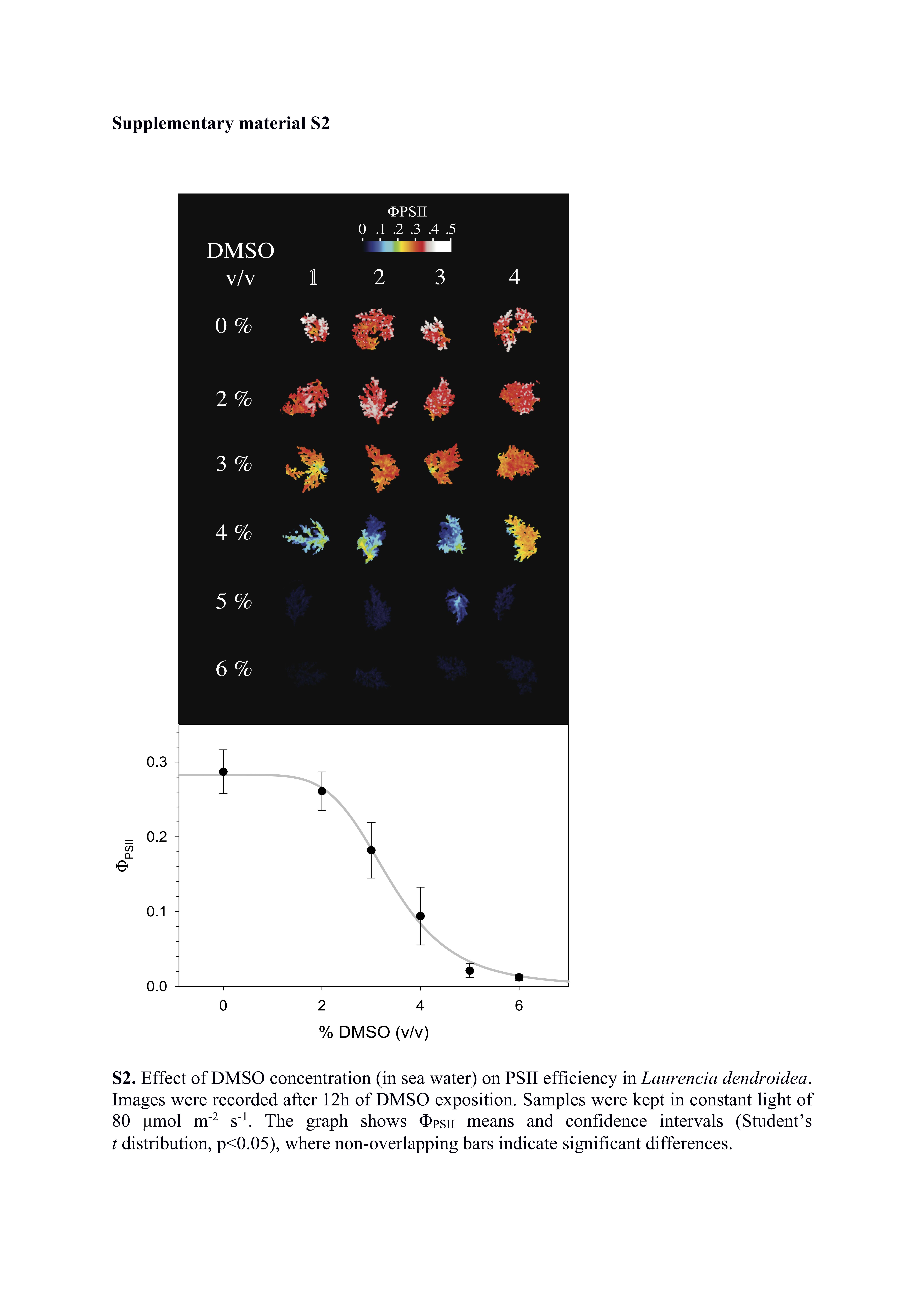

Supplement: Supplementary file 1 [file Image_1.jpg]

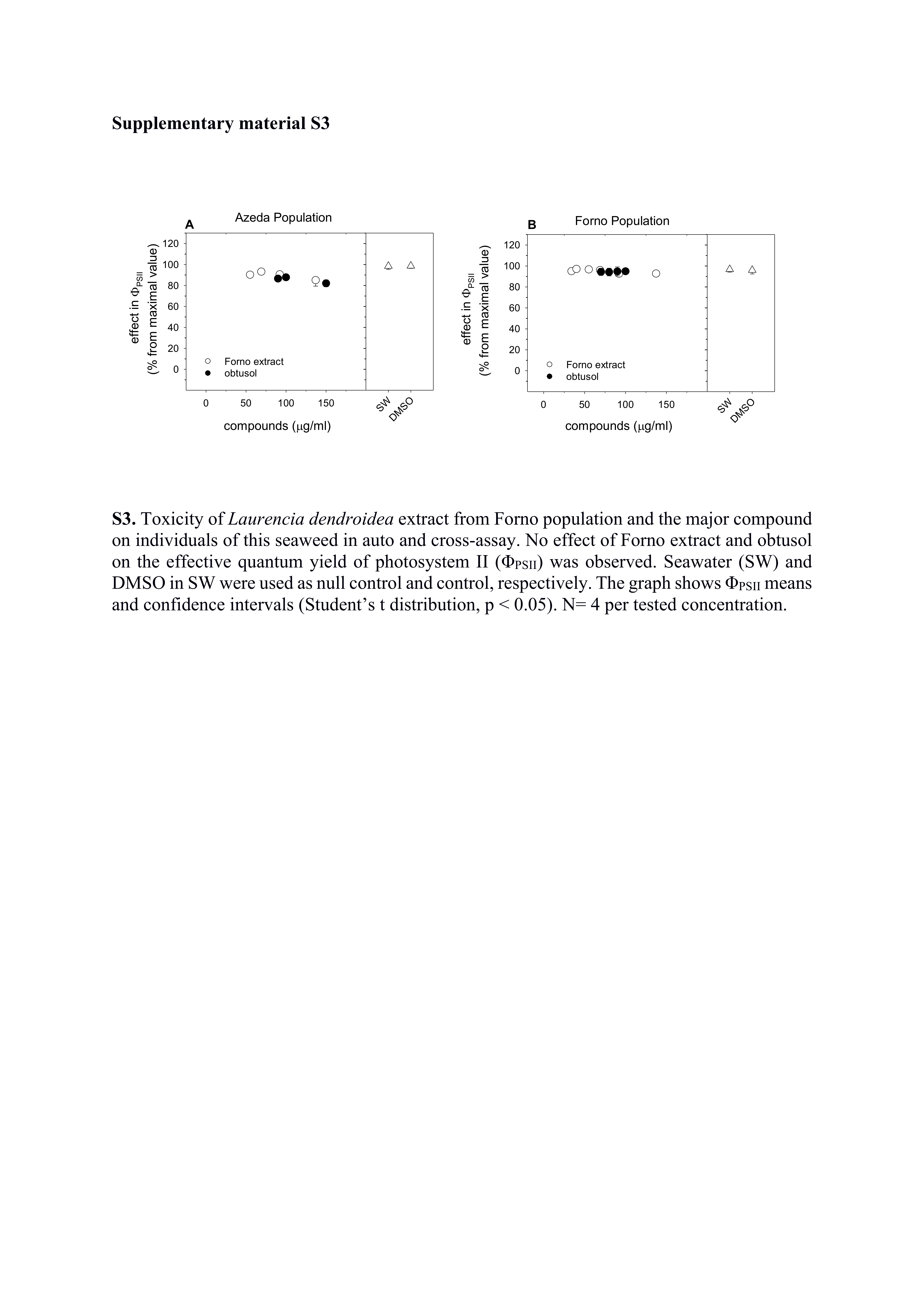

Supplement: Supplementary file 2 [file Image_2.jpg]
