# Supplementary material for: New Ecological Role of Seaweed Secondary Metabolites as Autotoxic and Allelopathic
Source: Front Plant Sci. 2020 May 25;11:347. doi: 10.3389/fpls.2020.00347 (PMC7261924; doi:10.3389/fpls.2020.00347)
Supplement: Supplementary file 3 [file Table_1.docx]

**Supplementary material S1**

**S1**. NMR ^1^H and ^13^C (CDCl_3_, 600 MHz and 300 MHz) data from (+)-elatol (I) and obtusol (II) in present study and reference literature.

|  | | | |  | | | | |
| --- | --- | --- | --- | --- | --- | --- | --- | --- |
|  | | | |  | | | | |
|  | | | | (+)- elatol | | | | |
|  | | | |  | | | | |
|  | | | | |  | | | |
| **(I)** CDCl_3_, 600 MHz | | | | KÖNIG & WRIGHT, 1977 | | | | |
| ***δ* ^13^C** | ***δ* ^1^H** | **m** | ***J* (Hz)** | ***δ* ^13^C** | | ***δ* ^1^H** | **m** | ***J* (Hz)** |
| 38,8 | 2,35 | d | 17,4 | 38,6 | | 2,08 | d | 17,5 |
|  | 2,57 | d | 17,6 |  |  | 2,19 | d | 17,5 |
| 128,2 | - |  |  | 128,0 | | - |  |  |
| 124,2 | - |  |  | 124,1 | | - |  |  |
| 29,5 | 1,81 | d | 11,8 | 29,3 | | 1,82 | m |  |
|  | 1,94 | d | 16,4 |  |  | 1,96 | m |  |
| 25,7 | 1,61 | m |  | 25,6 | | 1,62 | m |  |
|  | 1,81 | d | 11,8 |  |  | 1,80 | m |  |
| 49,3 | - |  |  | 49,1 | | - |  |  |
| 140,9 | - |  |  | 140,7 | | - |  |  |
| 38,1 | 2,49 | d | 14,5 | 38,0 | | 2,49 | dd | 2,8 – 14,4 |
|  | 2,62 | d | 14,6 |  |  | 2,19 | dm | 14,4 |
| 72,3 | 4,14 | d | 2,3 | 72,1 | | 4,14 | m |  |
| 71,0 | 4,60 | d | 2,3 | 70,8 | | 4,61 | d | 2,9 |
| 43,3 | - |  |  | 43,1 | | - |  |  |
| 21,0 | 1,05 | s |  | 20,7 | | 1,06 | s |  |
| 24,3 | 1,06 | s |  | 24,2 | | 1,07 | s |  |
| 116,1 | 4,78 | s |  | 115,8 | | 4,79 | s |  |
|  | 5,11 | s |  |  |  | 5,12 | s |  |
| 19,6 | 1,69 | s |  | 19,4 | | 1,70 | s |  |

|  |  |  | **** |  |  |  |  |  |
| --- | --- | --- | --- | --- | --- | --- | --- | --- |
|  |  |  |  |  |  |  |  |  |
|  |  |  |  |  |  | obtusol | | |
|  |  |  |  |  |  |  |  |  |
|  |  | | | | | | | |
|  | **(II)** CDCl_3_, 300 MHz | | | | GONZÁLEZ, *et al*. 1979, WESSELS, *et al.* 2000 | | | |
| **nº C** | ***δ* ^13^C** | ***δ* ^1^H** | **m** | ***J* (Hz)** | **δ ^13^C** | **δ ^1^H** | **m** | **J (Hz)** |
| **1** | 25,8 | 1,75 | m |  | 25,6 | SA |  |  |
|  |  | 178 | m |  |  |  |  |  |
| **2** | 40,7 | 2,29 | m |  | 40,5 | SA |  |  |
|  |  | 2,18 | m |  |  |  |  |  |
| **3** | 67,7 | - |  |  | 67,6 | - |  |  |
| **4** | 68,3 | 4,71 | dd | 1,8 - 10,4 | 68,1 | 4,70 | dd | 4,4 – 11,2 |
|  |  |  |  |  |  |  |  |  |
| **5** | 37,3 | 1,89 | d | 3,9 | 37,1 | SA |  |  |
|  |  | 1,96 | d | 12,9 |  |  |  |  |
| **6** | 50,3 | - |  |  | 50,3 | - |  |  |
| **7** | 141,2 | - |  |  | 141,2 | - |  |  |
| **8** | 38,7 | 2,62 | dd | 3,6 - 13,7 | 38,5 | 2,62 | dd | 3,1 – 14,2 |
|  |  | 4,50 | dd | 2,4 - 14,2 |  | 2,49 | dd | 2,4 – 14,2 |
| **9** | 71,8 | 4,12 | m |  | 71,9 | 4,11 | m |  |
| **10** | 72,1 | 4,47 | d | 2,8 | 70,1 | 4,47 | d | 3,0 |
| **11** | 44,4 | - |  |  | 44,2 | - |  |  |
| **12** | 24,3 | 1,08 | s |  | 24,2 | 1,08 | s |  |
| **13** | 20,3 | 1,08 | s |  | 20,6 | 1,08 | s |  |
| **14** | 118,0 | 5,05 | s |  | 117,8 | 5,05 | s |  |
|  |  | 5,39 | s |  |  | 5,39 | s |  |
| **15** | 24,1 | 1,83 | s |  | 23,9 | 1,83 | s |  |

**MS** (70eV) data: **(+)-elatol**

**
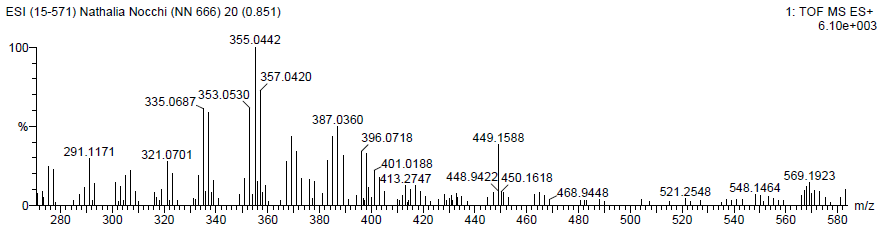
**

**HRMS-ESI**: **(+)-elatol**


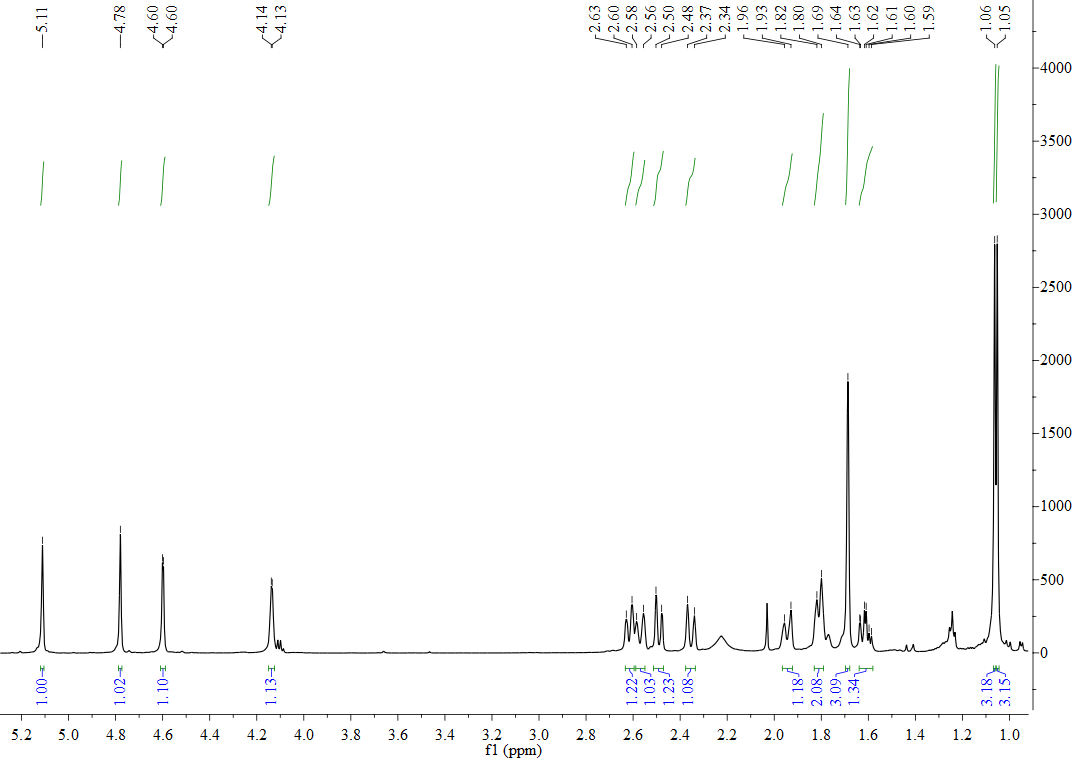


**H^1^ NMR** (600 MHz, CDCl_3_): **(+)-elatol**


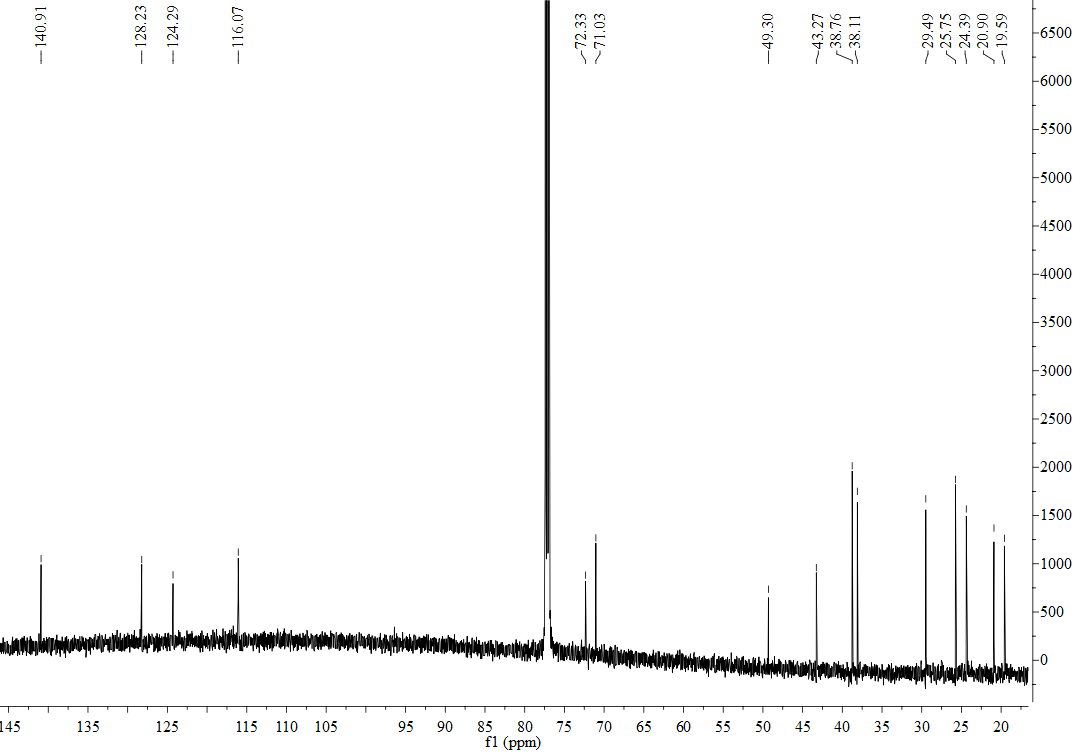


**^13^C NMR** (600 MHz, CDCl_3_): **(+)-elatol**

**MS** (70eV): **obtusol**


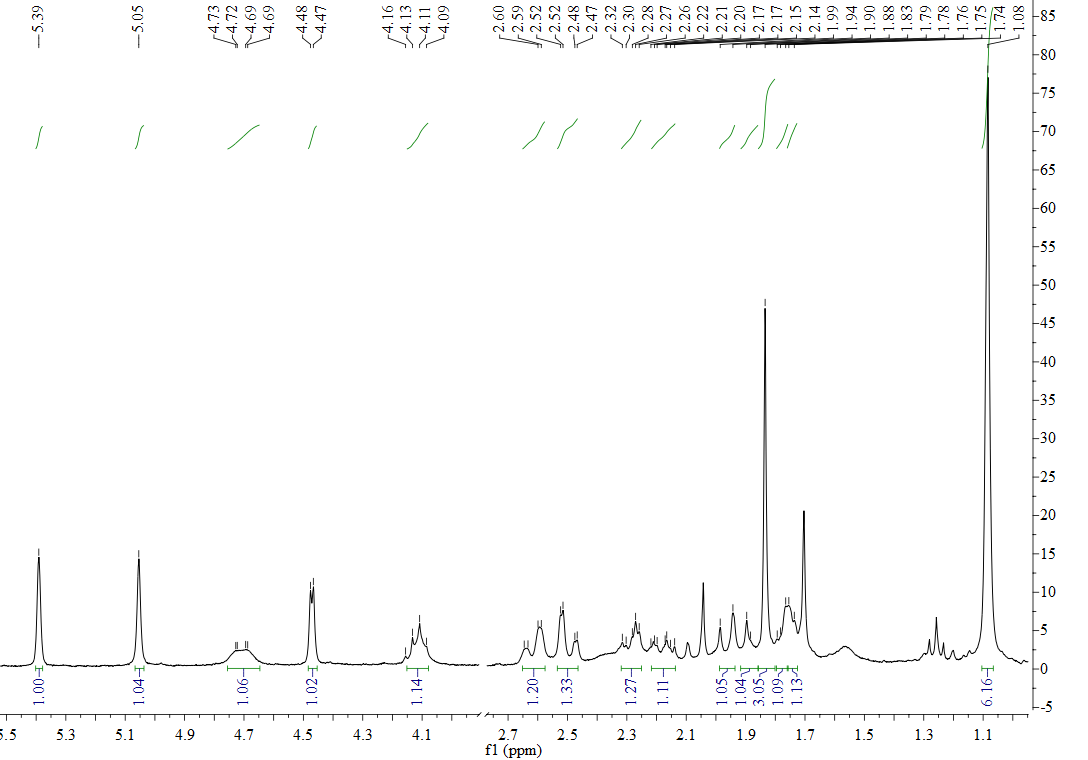


**^1^H NMR** (300 MHz, CDCl_3_): **obtusol**


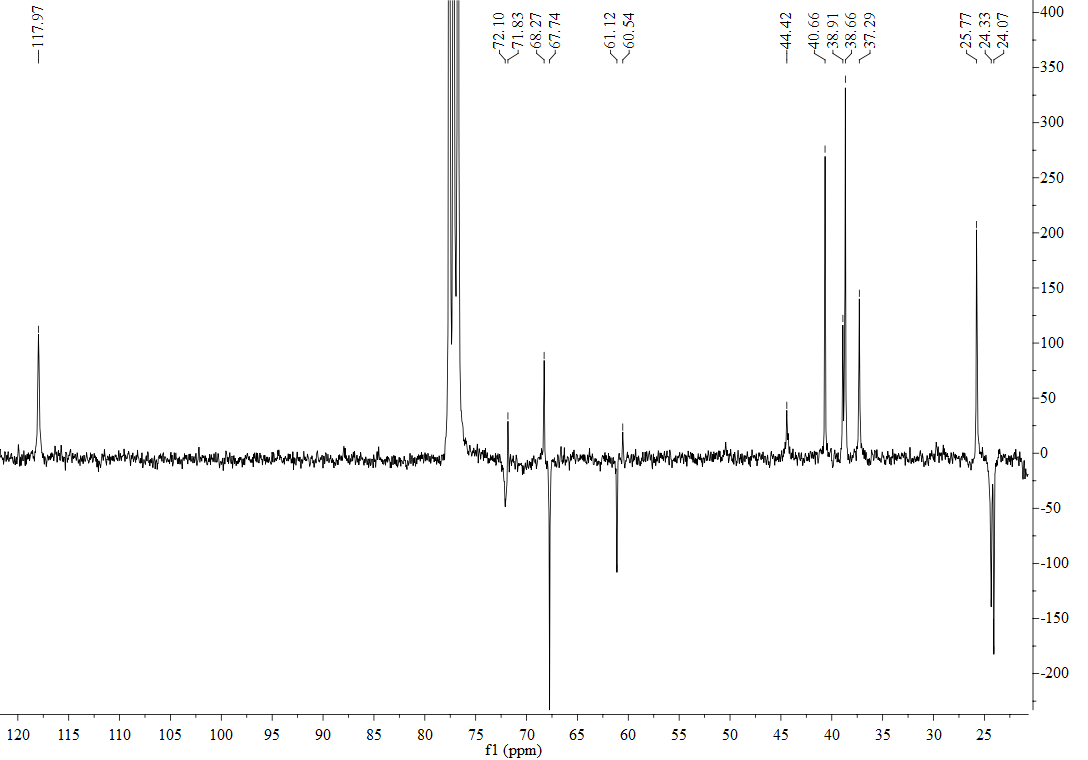


**APT NMR** (300 MHz, CDCl_3_): **obtusol**
